# Representation of Dynamical Stimuli in Populations of Threshold Neurons

**DOI:** 10.1371/journal.pcbi.1002239

**Published:** 2011-10-20

**Authors:** Tatjana Tchumatchenko, Fred Wolf

**Affiliations:** 1 Department of Nonlinear Dynamics, Max Planck Institute for Dynamics and Self-Organization, Goettingen, Germany; 2 Bernstein Center for Computational Neuroscience Goettingen, Goettingen, Germany; 3 Collaborative Research Center 889 Cellular Mechanisms of Sensory Processing, Goettingen, Germany; Université Paris Descartes, Centre National de la Recherche Scientifique, France

## Abstract

Many sensory or cognitive events are associated with dynamic current modulations in cortical neurons. This raises an urgent demand for tractable model approaches addressing the merits and limits of potential encoding strategies. Yet, current theoretical approaches addressing the response to mean- and variance-encoded stimuli rarely provide complete response functions for both modes of encoding in the presence of correlated noise. Here, we investigate the neuronal population response to dynamical modifications of the mean or variance of the synaptic bombardment using an alternative threshold model framework. In the variance and mean channel, we provide explicit expressions for the linear and non-linear frequency response functions in the presence of correlated noise and use them to derive population rate response to step-like stimuli. For mean-encoded signals, we find that the complete response function depends only on the temporal width of the input correlation function, but not on other functional specifics. Furthermore, we show that both mean- and variance-encoded signals can relay high-frequency inputs, and in both schemes step-like changes can be detected instantaneously. Finally, we obtain the pairwise spike correlation function and the spike triggered average from the linear mean-evoked response function. These results provide a maximally tractable limiting case that complements and extends previous results obtained in the integrate and fire framework.

## Author Summary

Sensory stimuli in our environment are represented in the brain as input current changes to neurons. For example, a periodic bar pattern in the visual field leads to periodic current modulations in the visual cortex. Therefore, models describing the ability of neurons to represent incoming stimuli can offer important clues about how sensory stimuli are processed by the brain. As anyone who has used an old-fashioned radio can attest, there is not just one but multiple ways to encode a signal, e.g. the familiar AM and FM channels. But what are the potential encoding channels in the cortex? A signal could modify the neuronal input current in two distinct ways: it could act either on the mean or the variance of the current. Using a minimal model framework, which can reproduce many features of neuronal activity, we find that both encoding schemes could be equally potent in transmitting slow and fast signals. This allows us to describe how input signals of any functional form give rise to collective firing rate changes in populations of neurons.

## Introduction

Intracellular recordings of multiple neurons have shown that dynamical sensory stimuli can modulate input currents to cortical neurons. For example, visual stimulation with moving gratings can lead to oscillatory modulation of the membrane potential in visual cortical neurons [1]. While it is important to identify the role such dynamic modulations play in neural coding [2]–[4], it is also important to understand how their encoding depends on physiological parameters such as the background noise or the firing rate. The incoming external signals can be encoded in the mean or variance of the synaptic current to each neuron in a cortical network. In a single neuron the maximal firing rate limits the highest faithfully encoded frequencies. Yet the spike rate is remarkably low, often below 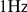 in cortical neurons [5], [6]. Therefore, the representation of perceptually important fast-varying stimuli has to emerge at the population level. In neuronal populations the frequency response function quantifies the fidelity of signal representation [7]–[10]. Intuitively, the frequency response function measures how well the population firing rate is modulated by the incoming signal of a specific frequency. If the amplitude of the rate modulation is zero then that frequency cannot be encoded in the population rate.

An early study by Knight showed that a population of independent (perfect) integrate and fire neurons can faithfully encode any input frequency, but if finite memory is introduced to the single neuron dynamics, the population rate is no longer a perfect copy of the stimulus [7]. Subsequent studies established that two factors play a particularly important role for the frequency response: the noise statistics and the spike generation mechanism [9]–[13]. Brunel and colleagues have shown that in the leaky integrate and fire model high-frequency mean-modulating signals are represented faithfully in the population rate only on the background of colored noise [10]. Substituting colored for white noise background, on the other hand, leads to 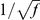 decay of the frequency function for input frequencies 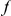 much larger than the stationary firing rate [9], [14]. The analytical complexity of the colored noise results, however, necessitates a largely numerical treatment and generally allows for explicit expressions in specific limits only, e.g. the linear regime of weak amplitudes [10], [15]. One notable exception are the recent results obtained by Ostojic, Richardson and colleagues for the non-linear response functions of the exponential integrate and fire model 16,17. Notably for variance modulations, the leaky integrate and fire model neurons can faithfully encode any high-frequency input even for white noise [9], [14]. So far, only specific limits of the frequency response function could be calculated in the integrate and fire framework for a limited set of temporal correlation functions. The linear response for mean modulating signals has been obtained analytically in the limit of white or almost white Ornstein Uhlenbeck currents by Brunel and colleagues in the leaky integrate and fire model [10]. Also Brunel and Latham obtained a linear response for mean modulating signals in the limit where the correlation time is much larger or much smaller than the membrane time constant in the quadratic integrate and fire model [18]. The high-frequency limit of the response function for mean modulations has been studied in various integrate and fire type models. Brunel and colleagues obtained in the exponential and quadratic integrate and fire model the response amplitude in the high-frequency limit and showed that it decay 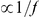 for frequencies 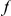 beyond the cut-off determined by the inverse spike initiation time [11], [15], [16], [18], [19]. More generally, integrate and fire type models with a variable spike onset initiation time have shown that for white as well as colored noise the representation of high-frequencies in the mean channel is successively enhanced if the spike onset time is reduced [11], [20]. For the variance modulation, however, the analytical results appear much more sparse in the literature and are available so far only in the white noise limit and in the linear regime of the leaky integrate and fire model [9], [21] or the perfect integrate and fire model [22]. In summary, these previous model studies have shown that the structure of the noise background can fundamentally change the response properties- particularly the difference between a perfect white noise and colored noise can be profound. The sharpness of the spike onset has little effect on the low and intermediate frequencies but strongly determines the high-frequency cut-off above which the decay of the frequency response function sets in [11], [12], [20]. Notably, recent experimental evidence indicates that cortical neurons can indeed encode input frequencies that are tens of times faster than the firing rate of individual neurons, in both mean- and variance-encoding schemes in the presence of *in vivo*-like correlated background noise [23]–[25]. This suggests that a threshold-based model that is driven by different types of colored noise can be a promising starting point to understand the fundamental determinants of the frequency response function for the physiologically important intermediate frequency range up to a few hundred hertz [23]–[25].

Here, we show that an alternative threshold-based framework can be used to obtain explicit and tractable results for the linear as well as non-linear response to mean- and variance-encoded stimuli. The explicit results derived here for the frequency response function, pairwise spike correlations and spike triggered averages constitute a maximally tractable limiting case that complements and extends the results obtained in the integrate and fire framework. Importantly, this framework does not limit the accessible current correlation functions to white noise or Ornstein Uhlenbeck process, thereby allowing us to explore a wide variety of shapes and time constants that can occur *in vivo* [26]–[28].

The manuscript is organized as follows: we start with the introduction of the mean and variance signaling in cortical networks in Section “Signal representation in cortical networks”. We then introduce the population firing rate response dynamics in Section “Key definitions of dynamical population response”. Subsequently, we define the model setting and compute basic quantities such as the firing rate of individual neurons in Section “Dynamical response in threshold model neurons”. In Section “Population response to mean and variance oscillations in the threshold model” we obtain the population rate dynamics in response to oscillatory changes of mean and variance. In Section “Response to step-like input current changes” we address the population response to step-like input changes. In Section “Weak pairwise spike correlations” we quantify the spike correlations in two neurons that are subject to a common fluctuating mean signal. Finally, we focus on the statistics of spike triggering events in Section “Spike-triggering events”. In discussion section we present a discussion of our results and their relation to previous theoretical and experimental findings. A nomenclature overview can be found in Table 1.

**Table 1:** Symbol nomenclature in the order of appearance.

Symbol	Description
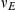	Excitatory input firing rate of a neuron in a balanced network
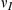	Inhibitory input firing rate of a neuron in a balanced network
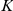	Average number of synaptic inputs
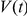	Voltage of a single neuron
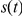	Spike train of a neuron
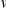	Firing rate of a neuron
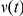	Time dependent population firing rate
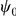	Threshold voltage
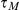	Membrane time constant
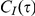	Current auto correlation in a single neuron
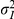	Current variance in a single neuron
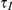	Temporal width of current correlation function
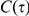	Voltage auto correlation in a single neuron
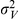	Voltage variance in a single neuron
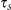	Temporal width of voltage correlation function; for Ornstein Uhlenbeck drive 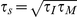
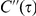	Correlation function of 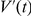 , equal to the second derivative of 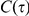
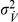	Variance of 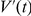 , equal to 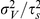
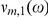	Mean evoked linear response function
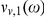	Variance evoked linear response function
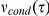	Conditional firing rate of two neurons
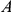	Signal amplitude
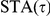	Spike triggered average

## Results

### Signal representation in cortical networks

**Figure 1:**
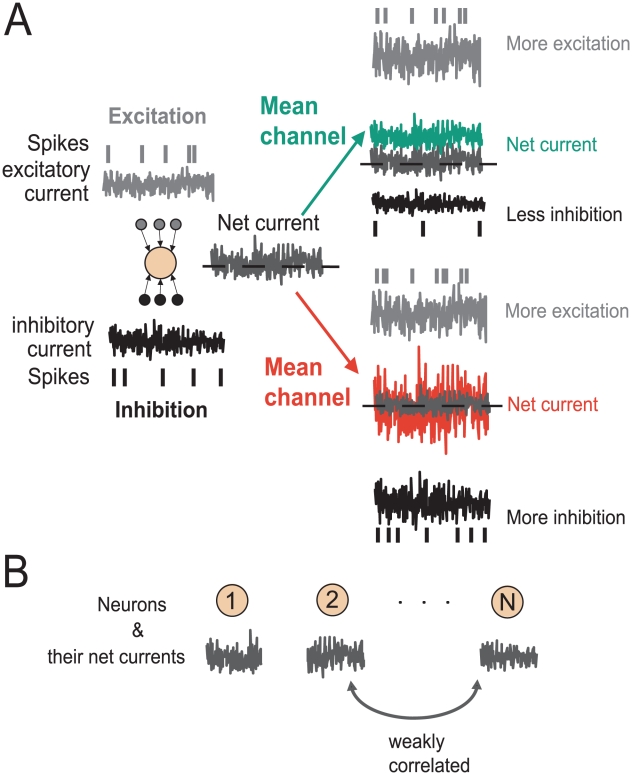
Encoding in the mean and variance channel. (A) Simultaneous increase of excitatory and reduction of inhibitory activity (or vice versa) results in a mean current change (right, green). On the other hand, simultaneous increase (or reduction) in excitatory and inhibitory spiking activity results in modifications of the net current variance (left, red). These modifications constitute two primary channels of communication in a cortical network. (B) In a cortical network the excitatory and inhibitory currents add up such that the net somatic current is only weakly correlated across neurons [31], [32].

Neurons in the mammalian cortex form an interconnected network, where each neuron receives inputs from many thousands of presynaptic neurons. The excitatory and inhibitory inputs at each neuron counteract each other [29], [30]. What results is an excitation-inhibition balance which is schematically illustrated in Fig. 1 A(left). In Fig. 1 A (left) the mean excitatory (grey) and mean inhibitory (black) currents are counteracting each other and result in a zero-mean net current at the soma of a neuron. Yet, the subtraction of excitatory input by inhibition is not perfect and the remaining net current has a sizable variance. What could be the benefit of operating in this way? Theoretically, it is understood that a neuronal population in such a state could encode and relay incoming signals via two channels (1) modifications of the mean synaptic bombardment and (2) modification of the synaptic fluctuation variance. These two encoding channels are schematically illustrated in Fig. 1 (A). In case (1) the signal is added to the input of neurons, in case (2) the signal modulates the variance of the background fluctuations in neurons, similar to the amplitude modulation strategy which is widely used in radio communication. To employ strategy (1) a sensory stimulus could alter the mean current by adding an external signal to the network generated background fluctuations. On the other hand, in a cortical population where the fluctuations in the activity of excitatory and inhibitory populations accurately track each other [30], [31] the effect of excitation and inhibition can be precisely balanced. In this case, any perturbation would result only in a change of input variance to each neuron and a balance of inhibition and excitation would compensate any mean current changes. To rapidly encode modulations of the input current in the population firing rate, the strategy employed by neurons needs to be susceptible to subtle changes of either mean or variance. Now let us formalize the definitions and define the mean and variance of net input currents in a cortical network in the absence of external signals. According to the calculations outlined in Methods Section “Current mean and variance in a cortical network” the input current mean and variance for all neurons in a cortical network can be described by:
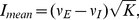
(1)

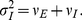
(2)In the mean channel, the activity of excitatory neurons needs to be increased by 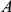 while the activity of inhibitory neurons is decreased by the same amount 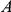. Fig. 1 (right, green) illustrates that this procedure modifies the mean but leaves the variance of the net current unaffected.
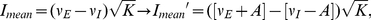
(3)

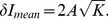
(4)

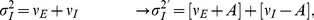
(5)

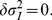
(6)In a second independent encoding scheme, a signal is encoded in the variance of the net current. As demonstrated in Fig. 1 (right, red) this could be achieved by increasing the excitatory and inhibitory firing rates simultaneously by an amount 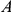:
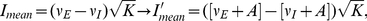
(7)

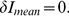
(8)

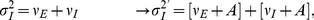
(9)

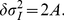
(10)It is plausible to assume that in live cortical networks, both mean and variance signaling act simultaneously. For the physiologically plausible regime of small but simultaneous mean and variance changes, the resulting population response is simply a superposition of the mean and variance responses. For large simultaneous modulations of mean and variance, the resulting population response needs to be computed on a case-by-case basis, because it can potentially depend on the stimulus form, and the amplitude ratio of mean and variance signals.

#### Effective independence assumption for cortical populations

To understand how interconnected cortical populations respond to mean or variance signals, two ingredients are crucial: 1) response of independent neurons and 2) the influence of topology. Before 2) can be addressed, the response of independent cortical neurons in 1) needs to be clarified. Notably, several recent studies have shown that the net input current and spike correlations observed *in vivo* cortical are predominantly weak, with spike correlation coefficients 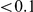 in awake animals [5], see Fig. 1. Additionally, recent studies have proposed active decorrelating mechanisms based on a precise temporal correspondence between excitation and inhibition in each neuron in a network, which result in pairwise decorrelated spiking activity [31], [32]. These exceptionally weak correlations suggest that the assumption of independent neurons can be a promising starting point for the investigation of response dynamics of cortical populations. Therefore, we focus in this study on the population firing rate dynamics of *independent* neurons subject to mean or variance modulating signals. The fluctuating background currents, which in a live network results from a sum of inhibitory and excitatory currents, will be synthesized independently for each neuron as a random realization of a random Gaussian process and modulated either in their mean or variance. In the following Sections we introduce the threshold-based single neuron model that we use to characterize the dynamical response in such a population of independently encoding neurons.

### Key definitions of dynamical population response

Before we start with the specific representation of dynamical signals in the threshold model, let us first specify model-independent definitions for the dynamical population response, such as linear response function or spike triggering events. A nomenclature summary can be found in Table 1.

**Figure 2:**
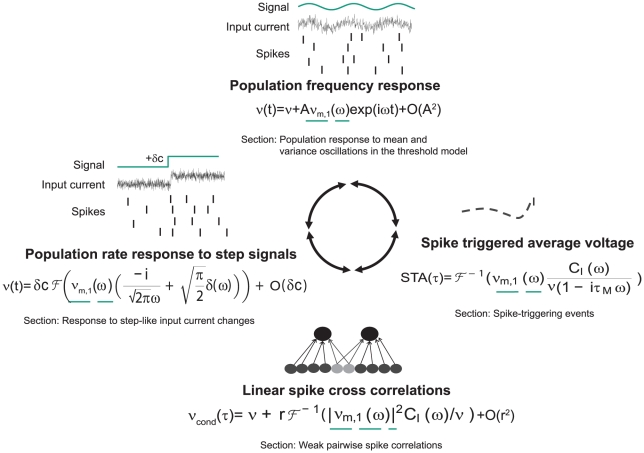
Computational role of mean-encoded signals. (Top) Representation of periodic mean stimuli in the population rate of noisy, independent neurons. (Left) Representation of step-like mean signals in the population rate of noisy, independent neurons. (Bottom) Common fluctuating currents from presynaptic partners represent a common mean signal that leads to pairwise spike correlation function 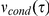. (Right) The average voltage before a spike is shaped by the linear mean response. 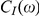 denotes the input current correlation function. The role of the linear response function 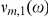 is indicated by a dashed green line. Results obtained in the alternative threshold model are discussed in the indicated Sections of this manuscript.

In the previous Section we established that signals in a cortical network can be encoded in the mean or in the variance of input currents. If this signal constitutes only a small perturbation of the system, as can be expected for example from thalamo-cortical projections [33], then the population firing rate 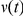 is directly proportional to the signal 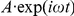:
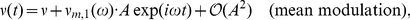
(11)

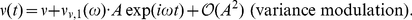
(12)The proportionality factors 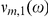 or 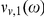 are referred to as the *linear response functions*. Each of these functions essentially describes how each signal frequency is affecting the firing rate. Note, that Eqs. 11 and 12 are model-independent, and that linear response dynamics for weak enough signals can be derived for any non-linear system. Also note that the *linear* response derives its name from the fact that the output rate 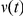 is linearly related to the input in Eqs. 11 and 12. Fig. 2 (top) schematically demonstrates the encoding of periodic mean signals in the presence of background noise. The resulting spikes collectively lead to a periodic modulation of the firing rate described by Eq. 11. In addition to the oscillatory changes in mean and variance another group of changes bears particular physiological significance. These are the step-like changes in mean and variance. To compute the population rate response to a step-like signal, we first formally describe that by a Heaviside 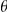-function:
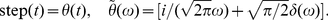
(13)

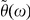
 is the Fourier transform of 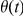. This model independent relation describes the contribution of each frequency to the step-like signal. To compute the neuronal response to the mean-encoded weak step-like stimulus, it is sufficient to consider each frequency separately, compute the respective output and sum up all contributions. The response to a step signal of size 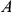 can then be formally written as a convolution:
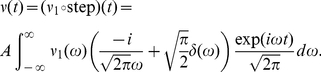
(14)In the case of mean channel and weak signals, the linear response function and its inverse Fourier transform 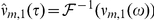 are the only functions [34] we need to predict the population response to *any* weak dynamical current stimulus 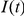:
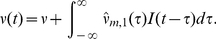
(15)For signals which constitute a larger dynamical perturbation, successively higher order Wiener kernels [34] may be necessary. Furthermore, the linear response function determines not only the response to dynamical signals but it also shapes the weak spike correlations in two statistically identical neurons driven by a fluctuating Gaussian current [34]:
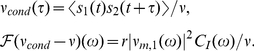
(16)Here, 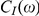 is the Fourier transform of the current correlation. The input current correlation strength is 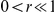. Eqs. 11–16 show that the response to variance and mean modulating signals are critical to a number of phenomena, from the processing of periodic stimuli to inter-neuron synchronization.

### Dynamical response in threshold model neurons

**Figure 3:**
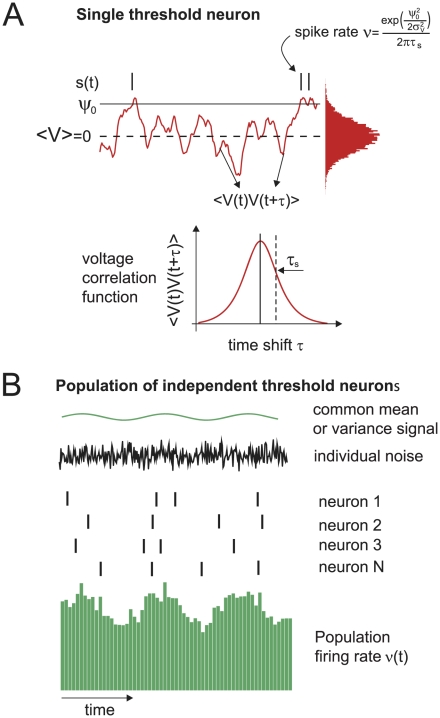
Spike generation and signal representation in the single spiking threshold neuron. (A) Spike generation from a temporally correlated Gaussian voltage trace in a single threshold neuron. (B) Encoding of common signals by the population firing rate 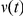 of independent threshold neurons. Note, that 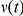 can be either linearly related to the stimulus (linear regime for weak signals, Eq. 11, 12) or be described by a non-linear response function (e.g. see Eq. 20).

Here, we use a previously introduced threshold-based model [35]–[38] that identifies the spike times 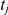 of a neuron with positive threshold crossings of a correlated, stationary Gaussian voltage 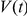 as it is illustrated in Fig. 3 (left):
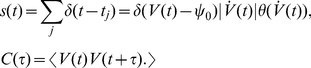
(17)

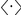
 denotes the ensemble average and 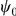 the spike threshold. A nomenclature overview can be found in Table 1. The voltage correlation function 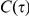 is characterized by its peak value 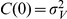 and the finite correlation time constant 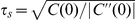. The correlation time 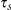, which is related to the autocorrelation time [37], describes the width of the correlation function in the vicinity of zero and is proportional to the zero crossings of a parabolic fit to the correlation function in the vicinity of zero. The second derivative of the correlation function 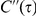 has a variance 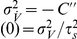. For simplicity we will assume a linear Resistor Capacitor (RC) membrane filter such that the Fourier transforms of the voltage and current correlation functions are related through 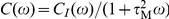. Our results, however, are not specific to this current voltage transformation, but can accommodate other filters that give rise to smooth voltage correlation functions [37], [38]. Note that this model does not have fixed-reset condition, but a silence period after each spike emerges from the regularity of the voltage trajectory [37]. The firing rate of a single threshold neuron is:
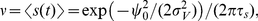
(18)where 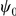 is the threshold voltage, 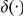 and 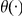 are the Dirac delta and Heaviside theta functions, respectively. In this approach the firing rate 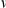 is a particularly tractable expression which depends only on two parameters: the correlation time 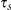 and the threshold-to-variance ratio 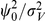, but not on the specific functional choice of the correlation function. Unless stated otherwise, we use the correlation function 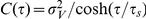 which is compatible with the power spectra of cortical neurons [26] for simulations using digitally synthesized Gaussian processes [39] or numerical integration of Gaussian integrals. Numerical simulations were implemented in Matlab 2010a (The MathWorks, USA) and analytical calculations were partially implemented in MATHEMATICA 5.2 and 8.0 (Wolfram Research Inc, USA). Let us note that the dependences on the threshold or current variance in Eq. 18 are consistent with the predictions in the leaky integrate and fire model, as we have shown in [37]. Note, that this threshold model operates only in the fluctuation driven, low firing rate 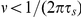 regime that is particularly important for visual cortical neurons [5], [6], [29], [30]. The mean-driven regime escapes the validity regime of this model.

#### Population response to mean and variance oscillations in the threshold model

**Figure 4:**
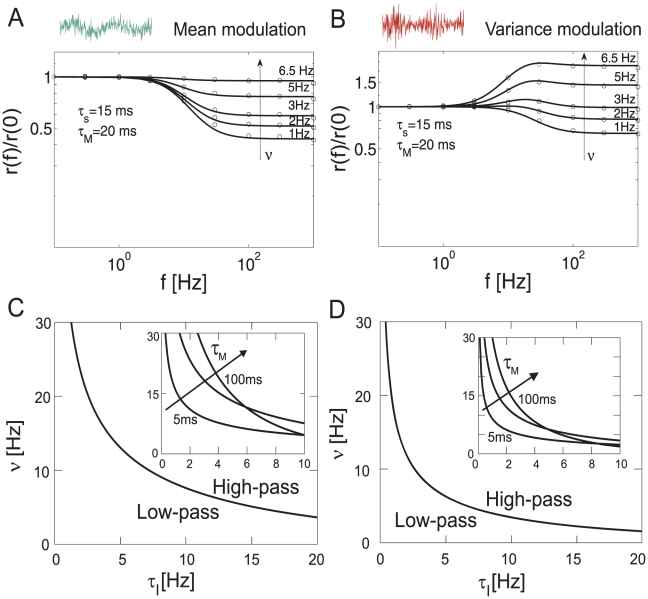
Linear response to mean and variance modulations in a population of independent threshold neurons. (A) Normalized amplitude 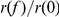 vs. 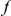 in response to mean current modulations, simulations (circles) and analytical results in Eq. 21 (solid line). (B) 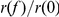 vs. 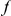 in response to current variance modulations, simulations (circles) and analytical results in Eq. 24 (solid lines). Regimes of high-pass and low-pass behavior for linear response function for mean (C) and variance modulations (D). Note, vector strength 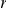 in (A) and (B) is proportional to the linear response 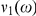, see Eq. 53.

**Figure 5:**
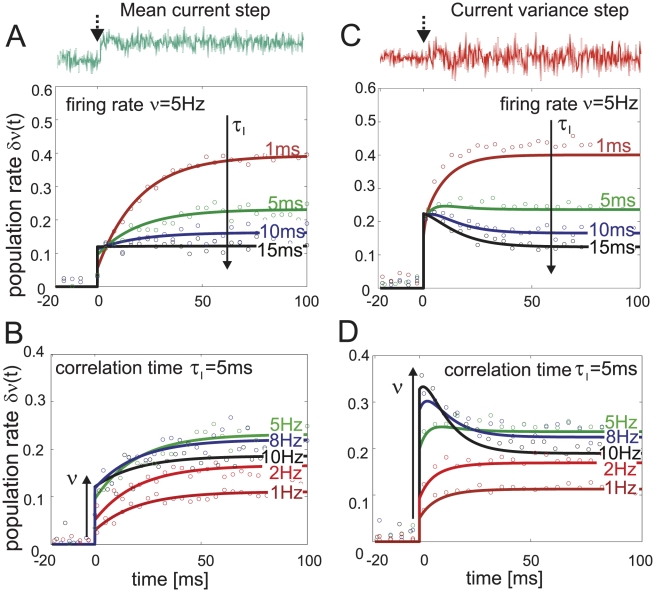
Firing rate response to a step-like current signal at time 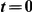
 **in a population of independent threshold neurons.** (A,B) Firing rate change 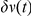 in response to a mean current step-like increase of amplitude 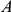, amplitude-to-threshold ratio 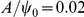. Analytical solution in Eq. 25 (solid lines) and simulation results (circles) are superimposed. (A) 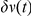 for 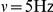, 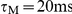 and varying current correlation times 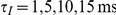. (B) 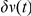 for 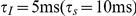, 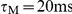 and 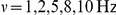. (C,D) Firing rate change 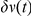 in response to an step-like increase of the current variance 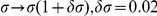. (C) 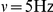 and 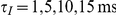, 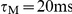. (D) 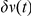 for 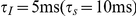, 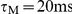 and 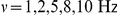. Analytical solution in Eq. 26 (solid lines) and simulation results (circles) are superimposed. Note that the evoked change of the stationary firing rate in A and C, B and D is the same.

**Figure 6:**
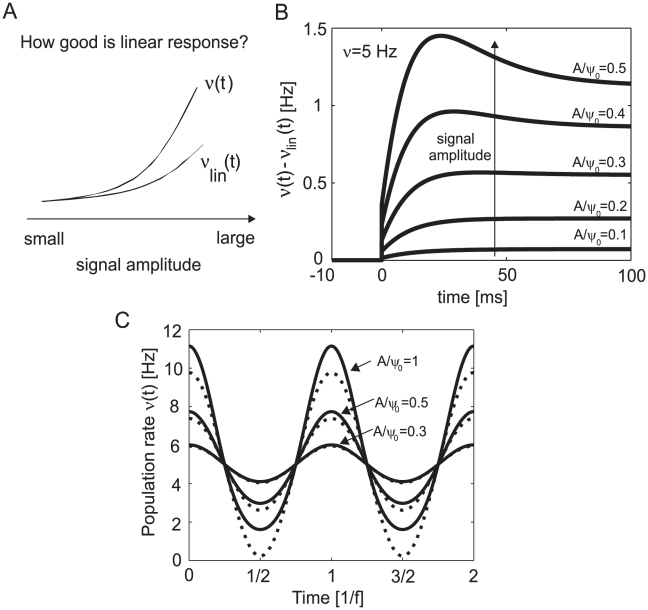
Fidelity of the linear approximation in relation to the complete non-linear response. (A) Schematic illustration of how well the linear approximation of the population rate derived for low amplitudes (as in Eqs. 25,26) captures the complete response dynamics. (B) Differences in linear and non-linear population firing rate in response to mean current steps of different amplitudes. Here, the linear response corresponds to Eq. 25 and the non-linear response derives from Eq. 20; stationary firing rate 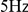 and 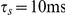. (C) Differences between the complete population firing rate (solid line, Eq. 20) and its linear approximation (dashed line, Eq. 21) in response to periodic mean modulations of 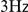 of different amplitudes. For an illustration of how the population dynamics 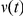 emerges in response to a dynamic stimulus see Fig. 3B.

We compute the population rate dynamics in response to changes of the mean and variance for the full range of input frequencies. First, we start with the mean modulations. As shown schematically in Fig. 3 (B) this paradigm subjects each neuron to a current which consists of a periodic signal and a fluctuating background background noise which is unique to each neuron. To calculate the response evoked by a sinusoidal modulation 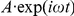 of the mean in the threshold model framework, the signal is low-pass filtered in the cell membrane:
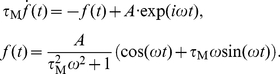
(19)Following the steps outlined in the Methods Section “Mean modulation”, Eq. 17 can be modified to accommodate the oscillating voltage-to-threshold distance. The complete non-linear population firing rate response reads:
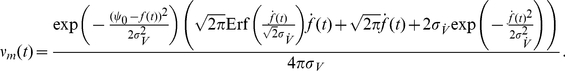
(20)Note that the firing rate modulation in response to periodic mean current is independent of a particular functional form of the current correlation function, because 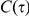 does not enter Eq. 20. We also find that Eq. 20 can be generalized to describe the population response to any signal 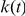. In this case the membrane filtered signal 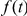 in Eq. 20 only needs to be replaced with the corresponding solution of the inhomogeneous differential equation in Eq. 19. From Eq. 20 we identify the linear mean evoked response:
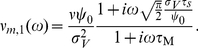
(21)Here, we find that the linear mean response is determined by only two time constants that shape the interplay between a low- and a high-pass. The amplitude of the linear response function for mean modulating signals in Eq. 21 is finite for any input frequency regardless of the stationary firing rate, see Fig. 4 (A). In the cross-over regime where the high-pass filter of the fixed threshold (nominator in Eq. 21) starts to counteract the low-pass filter of the membrane (denominator in Eq. 21) the response function transitions to a new firing rate dependent constant level. It is conceivable that the low-pass filtering by the membrane RC-circuit carries over to the firing rate dynamics, however, in this model even the highest frequencies can be relayed almost unattenuated. When is the linear approximation valid? As demonstrated in Fig. 4 and Fig. 5 linear response can be a good approximation for very small signal-to-threshold ratios of a few percent. In Fig. 6C we observe that for a signal-to-threshold ratio of 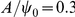 the linear approximation in Eq. 21 starts to be less accurate and non-linear effects kick. For 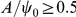 we observe that the linear approximation in Eq. 21 substantially overestimates the population response to hyperpolarizing (negative) transients and underestimates the response to depolarizing (positive) transients.

Now, we address the population rate response to periodic modulation of the current variance. The membrane filtered voltage signal 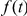 is then given by:
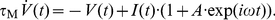
(22)Using 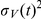, 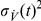 and 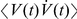 as given in the Methods Section in Eq. 48,49 and 51 we obtain the population firing rate:
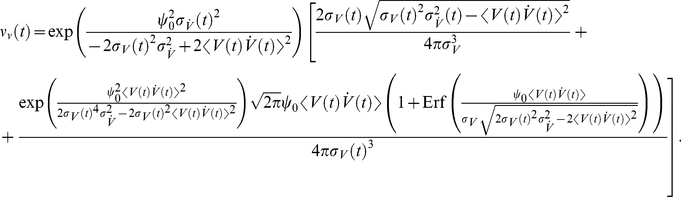
(23)The linear response function is:
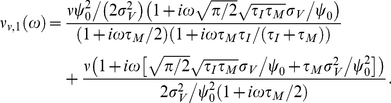
(24)Here, the 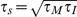 corresponds to the width of the voltage correlation function, 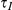 is the current correlation time, and 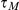 is the membrane time constant. Note, that Eq. 24 has been derived for an Ornstein Uhlenbeck current correlation of the form 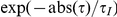. We find that Eq. 24 is characterized by an interplay of two high and low-pass functions each with an individual time constant. In the mean modulating channel, on the other hand, we identified only one high- and one low-pass, and only two effective time constants. Fig. 4 (B) shows that the response for variance modulating signals is finite for any frequency, regardless of firing rate. A comparison between Fig. 4 (A) and Fig. 4 (B) reveals that mean and variance modulation can relay both slowly and fast varying signals.

#### Response to step-like input current changes

Abrupt changes in input current statistics can convey the onset of a sensory stimulus. Therefore, the time it takes for a population of neurons to alter its firing rate can impose limits of the detection and operation speed of cortical rate encoding [11]. It is conceivable that the membrane low-pass filtering could carry over to the firing rate dynamics and lead to a detection time scale in the order of the membrane time constant. Here we show, however, that the threshold model defies this intuition and can signal instantaneously the onset of mean- or variance-encoded step-like signals, even if they are subthreshold. Using the linear response function for mean modulations 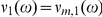 in Eq. 21 we obtain the following population rate change in response to step-like signals of amplitude 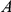:
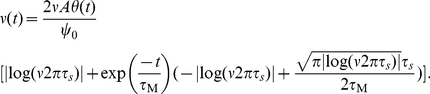
(25)Here, 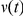 describes the population rate transient in response to a step-like increase of mean current 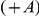 at 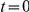. Eq. 25 and Fig. 5 (A,B) demonstrate that the response dynamics consists only of two components: an instantaneous component and an exponential governed by a single time scale 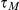. We recognize that a similar instantaneous component has been reported for Ornstein Uhlenbeck drive in the leaky integrate and fire model [10], but not for white noise drive [10], [14], [15]. For the white noise drive in the leaky as well as exponential integrate and fire model the response time scale is generally slower than the instantaneous component reported here. But their response time scale can, depending on input variance and firing rate, also be fast and substantially below the membrane time constant (see Fig. 1 F in [15]).

As the stimulus amplitude increases, the linear approximation in Eq. 25 breaks down and needs to be replaced by the corresponding full response dynamics in Eq. 20. Fig. 6 schematically illustrates the range of amplitude strengths for which the non-linear effects kick in. Fig. 6B shows that already at an amplitude-to-threshold ratio of 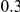 sizable deviations of 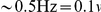 from the linear approximation are to be expected.

Now, we come to the population rate dynamics evoked by variance changes. Using the linear response function for variance modulations 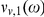 (Eq. 24) we obtain the population firing rate transient in response to a step-like increase 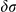 of the standard deviation:
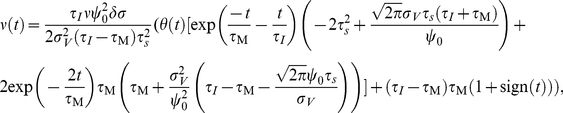
(26)where 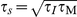. Fig. 5 shows the population rate transients in response to step-like changes in mean and variance predicted by Eqs. 25 and 26 alongside simulated results. Similarly to mean current steps, we find that for any 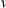 or 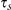 the variance evoked response dynamics consists of an instantaneous component and exponential transient. The magnitude of the instantaneous component is increasing with firing rate but is largely unaffected by the correlation time 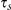. Thus, the response to mean and variance steps reported here is in line with the rapid response time observed *in vitro* cortical neurons [14], [25] and also with the instantaneous response observed in the leaky integrate and fire model driven by colored noise [9], [10], [21].

#### Weak pairwise spike correlations

**Figure 7:**
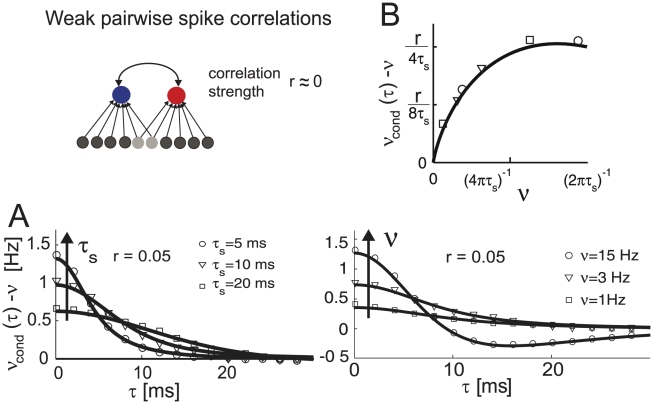
Weak spike correlations in the threshold model. (Top) Illustration of spike correlations resulting from common input that are studied in A and B. (A) Cross conditional firing rate 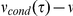 vs. time 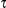 in the limit of weak input correlations 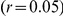. Both neurons have the same voltage correlation function 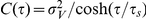, firing rate 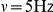. Fixed firing rate and varying correlation times 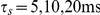 (A, left) or fixed correlation time 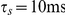 and varying firing rates 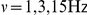 (A, right). (B) Peak spike correlation 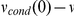 as a function of firing rate 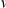. Symbols denote the corresponding peak spike correlations from (A).

The common fluctuating current component shared by two neurons in a network can be viewed as a superposition of different frequencies. As such, it can be analyzed using the same tools as oscillatory or step-like signals studied in the previous chapters. Before we start, let us briefly review why we include pairwise spike correlations among the most crucial phenomena shaped by response functions. In cortical ensembles, pairwise spike correlations are known to play an important role in influencing population encoding strategies [40] and even predicting multi-neuronal firing patterns at larger distances [41]. Here, we focus on linear, weak pairwise spike correlations of two neurons firing at the same rate 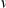. For simplicity, we assume the same statistical structure for the common fluctuating component 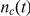 and the individual noise components 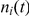 which make up the total input currents 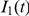 and 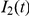 in the two neurons. To account for the weak input correlations [5], [31], [32] we assume a weak correlation strength 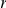, 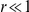, between the two input currents:
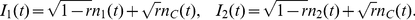
(27)The current correlation function 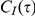 and the corresponding voltage correlation function 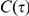 are the same for both noise components. We characterize the spike correlations via the conditional firing rate 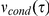:
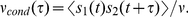
(28)Because the contribution of 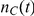 to the overall spiking activity is small and we can express it as a convolution of the linear impulse response function 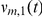 as in Eq. 15 [34]:
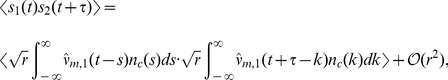
(29)

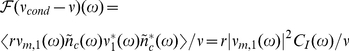
(30)

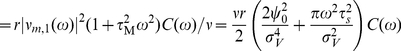
(31)

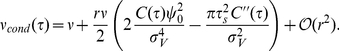
(32)The key ingredient in the calculation above is the property of the Fourier transforms of temporal derivatives, which for any function 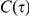 are 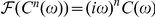. The peak value of this pairwise spike correlation is given by
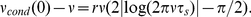
(33)
Fig. 7 illustrates 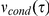 as a function of firing rate and time constant. We find that peak spike correlations increase with firing rate (Fig. 7 A) and decrease with increasing time constant 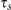. Furthermore, the firing rate dependence of spike correlations captured by Eq. 33 corresponds to the firing rate dependent increase reported in the leaky integrate and fire model driven by white and correlated noise [42], [43]. Furthermore, we can relate the correlated activity in Eq. 32 to the response dynamics evoked by oscillatory and step-like stimuli that we presented in previous sections. As we can see in Eq. 31 the firing rate dependent high-pass filter in Eq. 21 contributed the two terms proportional to 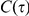 and 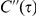 to the pairwise correlation function in Eq. 32. Therefore, we can conclude that the firing rate dependence of linear mean evoked response function 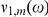 is directly related to the firing rate dependent shape of the pairwise spike correlations, in particular their firing rate dependent peak height and width.

#### Spike-triggering events

**Figure 8:**
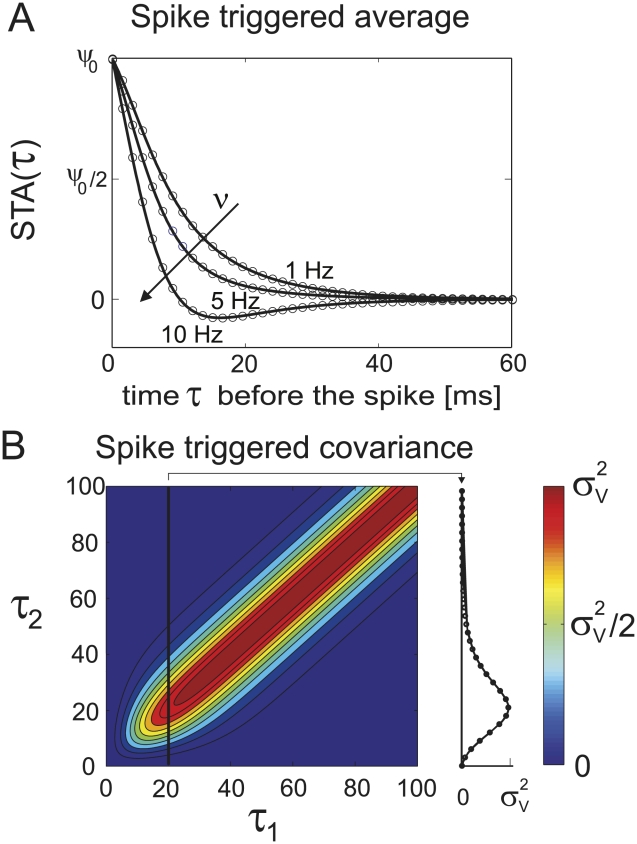
Statistics of spike triggering events in the threshold neurons. (A) Spike triggered average voltage 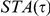 for 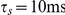 and firing rates 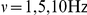; simulated results (circles) and analytical solution in Eq. 37 (solid lines). (B) Spike triggered voltage covariance 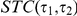 for 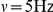, 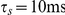, the cross section 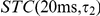 is shown at the right. Simulated results (circles) and analytical solution in Eq. 39 (solid lines). The solid vertical black line indicates 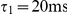.

The dynamical response explored in the previous chapters results from the collective spike decisions of many neurons. As such it is intimately linked to the spike times and the spike triggering events on the level of single neurons. Here we explore the link between the dynamical population response and the voltage events which lead to the spike decision in individual neurons. Let us first formally define the spike triggered average voltage 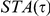, a time lag 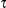 before a spike:
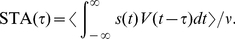
(34)Considering the spike train 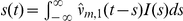 and the Fourier transform of the voltage correlation function 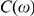 we obtain:
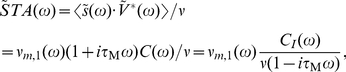
(35)

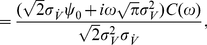
(36)

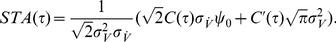
(37)The key ingredient in the calculation above is the property of the Fourier transforms of temporal derivatives, which for any function 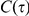 are 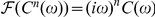. An alternative derivation of this result via the Gaussian integrals is also shown in the Methods Section “Spike triggered average voltage”. Fig. 8A demonstrates 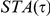 as a function of firing rate 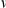. As expected, we find in Fig. 8 that the spike triggered average voltage is mostly increasing towards the spiking threshold as the time to the spike is reduced. We also recognize that the spike triggered average exhibits a firing rate dependent transient hyperpolarization which is more pronounced for higher firing rates. We also note that the increasingly pronounced hyperpolarizing transient emerges even in the absence of any oscillatory component in the voltage correlation function. Notably, the rate dependent hyperpolarizing transients prior to spikes have been previously observed in cortical neurons [44]. The more pronounced hyperpolarizing transient has been interpreted as the possibility that properly timed hyperpolarizing events may increase the firing probability, potentially through a reduction of sodium channel inactivation or spike frequency adaptation [44]. We find here, that the transient hyperpolarization is mediated by the second term in Eq. 37 that is proportional to 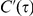. It originates from the high-pass contribution of the response function 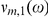. Let us also compare the result in Eq. 37 with the spike triggered average reported for the integrate and fire framework. Even though no analytical form exists for the complete spike triggered average in the integrate and fire model driven by colored noise, we can compare the large time limit 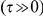 of Eq. 36 with the result reported by Badel and colleagues for the passive membrane. Eq. 17 in [45] reports 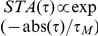 for an Ornstein Uhlenbeck current in the limit of low rates. Similarly, for very low rates we obtain via Eq. 36 that 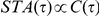. For a RC-filtered Ornstein Uhlenbeck current the voltage correlation function 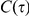 is a sum of exponentials where the longest time scale is 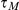, 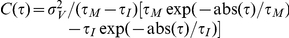. Taking this into account, we find for large 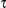 that 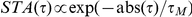 which is consistent with the integrate and fire result.

The spike triggered voltage covariance 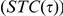 is an additional popular and easily accessible measure for the characterization of a neuronal model or live neurons *in vitro* [44], [46], [47]. It is related to the second order Wiener (e.g. see p.330 in [48]) and could therefore serve well in future model-model or neuron-model comparisons. Using calculations similar to those of Burak and coworkers [36] and standard Gaussian integrals we obtain STC in the threshold model:
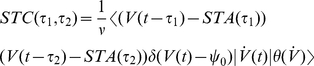
(38)

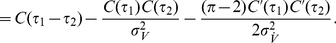
(39)If 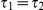 we find the spike triggered variance 
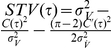
.

## Discussion

Here, we examined the relation between the frequency response functions in the mean and variance channels and the physiological parameters of single neurons, such as the functional form of the input current correlation, firing rate and membrane time constant. The threshold-based single neuron model we considered belongs to the class of spiking models that initiate a spike instantaneously after the threshold voltage is crossed. Instead of using the Fokker Planck framework we obtained the frequency response function via a direct integration of Gaussian probability densities that were modified to accommodate the mean or variance signals. This allowed us to systematically quantify the complete as well as linear frequency response in both channels, along with a number of related quantities such as population response to step signals, pairwise spike correlation function and spike triggered average. We confirmed all analytical results in numerical simulations of corresponding spiking neurons.

### Frequency response functions in the mean and variance channels

We derived the complete as well as linear frequency response function for mean modulating signals (Eq. 20,21). As can be expected from models with an instantaneous spike generation, the frequency response functions had a finite high-frequency limit. In the linear regime the mean evoked frequency response could be reduced to an interplay between a low-pass and a high-pass filter which were governed by only two independent time constants: the membrane time constant 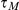 and an effective threshold-dependent time constant. Furthermore, both linear and non-linear response functions did not depend on the specific functional form of the voltage correlation function 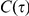, only its temporal width 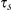 was important for the dynamical response. Notably, the population response to a mean current step could be described by an explicit tractable expression consisting of two components: the instantaneous component governing the immediate response and an exponential transient described by the membrane time constant (Eq. 25). We also found that the linear step-response function can be a good approximation for the population rate response if the signal-to-threshold ratio is below 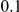.

For variance modulating signals we were also able to provide the complete as well as linear frequency response function (Eq. 23,24). We observed that the frequency response function remained finite in the high-frequency limit. In the linear regime, the variance evoked response could be described by an interplay between two high- and low pass functions. As in the mean channel, we found that population response exhibited two components: the instantaneous component that occurs immediately after the step onset and a combination of exponential functions that were governed by three time constants (Eq. 26). For the pairwise spike correlations that were obtained using the mean evoked linear response, we observed that the spike correlation peak increased and the temporal width decreased with firing rate (Fig. 7). The spike triggered average voltage and current could be described by an explicit expression as a sum involving the correlation function and its second derivative. Here, we found that with increasing firing rate the spike triggered average exhibited an increasingly pronounced undershoot shortly before a spike (Fig. 8 A).

### Model limits

The model framework that we used here to describe the spiking activity of each neuron in a cortical population is based on three major assumptions. The first is the confinement to the fluctuation-driven regime, second the Gaussian voltage statistics [49] and third the assumption that spikes are instantaneously generated upon the crossing of a fixed threshold voltage. The confinement of this model to the fluctuation-driven regime and Gaussian voltage statistics is in line with the recent experimental evidence that the fluctuation rather than mean depolarization driven regime is the primary operation scheme in cortical neurons. The first line of support is the remarkable cortical balance of excitation and inhibition such that neuronal firing is driven by fluctuations that transiently escape this cancellation [29], [30]. The second line of evidence are the exceptionally low firing rates 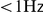 [5], [6]. Mean and fluctuation driven regimes can differ significantly in their spike train regularity, yet they seem to exhibit very similar spike cross correlation [42] and dynamical response properties [23]. Therefore, numerous features of dynamical population response could already be understood by studying only the fluctuation-driven regime. Even though skewed input current distributions that violate the Gaussian assumption can be of interest in specific cases, experimental evidence suggests that the Gaussian distribution can be a good match for *in vivo* background fluctuations in the prevalent cortical cell type of pyramidal neurons, e.g see Box 1 (top) in [26]. The third assumption, that spikes are instantaneously generated upon the crossing of a fixed threshold voltage, is motivated by the observation that time scales of spike onset can be very short, e.g. the onset rise time 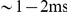 and the spike slope factor that is proportional to the radius of the curvature of the I–V curve at its minimum is 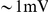 [50], [51]. As predicted by the previous models, the instantaneous threshold-based spike generation assumed in our model leads to a non-decaying frequency limit in the response functions [11], [19], [20]. These studies indicated that the frequency response function for low and intermediate frequency range are largely unaffected by the spike onset time and their properties can be could be explored in a model with an instantaneous spike generation mechanism. Alternative neuron models with more involved spike generation mechanisms such as quadratic or exponential integrate and fire indicate the possibility that the frequency response functions in a pair of neurons can depend on the model specifics [15], [52]. Yet, realizing how remarkably accurate many cortical spike synchrony features and response dynamics can be modeled by a clearly barebone-threshold model, we are convinced that this model will find its place alongside the classical integrate and fire models and offer a valuable maximally tractable limiting case for future studies.

### Comparison with previous theoretical studies

The linear and non-linear response functions are basic tools for the description of any physical system. In theoretical neuroscience several earlier studies have quantified the response functions in various model types, particularly in the integrate and fire models. To our knowledge, our study is the first to provide explicit expressions for the complete linear and non-linear response functions for both mean and variance channels in the presence of correlated noise, and also to derived from them tractable expressions for the step-evoked population response function, the full pairwise spike correlation function and the spike triggered averages. In the mean channel we showed for the first time that the frequency response function and all derived quantities can be independent of the functional form of the input correlations. The only important parameter determining in our formalism the frequency response function, step response dynamics or correlation gain was the width of the correlation function, but not its functional specifics.

Brunel and colleagues showed analytically in the integrate and fire model driven by the correlated Ornstein Uhlenbeck current that the linear frequency response function has a finite high-frequency limit [10]. Furthermore, the authors obtained numerically the functional form of the linear response function and the corresponding step-evoked transient and observed that two factors increase the high-frequency response and the instantaneous component of the step response: the firing rate and the temporal width of the correlation function. In our threshold model, we observe the same functional dependence of the linear response function on firing rate and temporal width. Here, however, we could show that this behavior is not unique to the Ornstein Uhlenbeck current. In fact, these effects can be observed for a wide range of colored input because the functional form of input correlation does not influence the frequency response function. This is important news, because it indicates that results obtained for the Ornstein Uhlenbeck current in the integrate and fire model [10], [15], [21], [52] could also be valid for more general current correlation functions. Let us also note, that the instantaneous response component in the leaky integrate and fire model vanishes for the physiologically remote choice of white noise drive (see Fig. 3 (top left) in [10]). In this case, the response dynamics is a superposition of different time constants [10], [15]. Therefore, the presence or absence of the instantaneous response component is model specific and some models such as the exponential integrate and fire lack an instantaneous component, but their response time is finite and can be faster than the membrane time constant (Fig. 1 F in [15]).

In the variance channel, Lindner and colleagues showed that the leaky integrate and fire model supports a faithful transmission of high-frequency inputs for white noise drive, see Eqs. 4–6 [9]. Also a number of follow-up studies confirmed this in the leaky, quadratic and exponential integrate and fire models [9], [12], [14], [19], [21]. However, these results focused on white noise background and provided the linear response function for colored noise only in specific limits, such as the infinitesimal or infinite input frequency limit [19] and did not address the full linear or non-linear response functions. Therefore, Eqs. 23 and 24 are, to our knowledge, the first to describe the complete as well as linear frequency response function in the presence of correlated noise. Also for the step response dynamics in the variance channel, only the slope of the initial response phase could be obtained so far, e.g. see Eq. 28 in [19]. Here, however, we provide the complete step-evoked response function Eq. 26 that can be generalized to the non-linear response regime using Eq. 24.

### Relation to experimental data

Starting in 1970s a number of studies have addressed the frequency response functions in cortical neurons. The majority of these studies [23], were conducted in the mean channel under a variety of different noise conditions. Bair and colleagues have shown that neurons in the medial-temporal (MT) area of the monkey cortex reliably transmitted input frequencies in the range 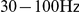 [55]. Also, three subsequent experimental studies in more controlled *in vitro* conditions have demonstrated that the reliably encoded frequency range can be tens of times larger than the firing rate of individual neurons. For a firing rate of 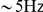 this can mean a reliably encoded frequency rage of up to 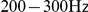 [23]–[25]. This is consistent with broad reliably encoded frequency range of the threshold model presented here, as well as the integrate and fire model for colored noise. Notably, the class of models with threshold-based instantaneous spike initiation are so far the only model types that enable reliable transmission of inputs that are much higher than the firing rate of individual neurons [11], [19], [20]. A key, prediction of our model framework is that response to high frequencies in the mean channel can be enhanced by a higher correlation time of the noise background. Notably, this correlation time dependence of the response function has been shown *in vitro* [25]. Here, we conclude that essential properties of the mean evoked frequency response function can be understood within the results derived from the threshold model. In fact, this model goes a step further and predicts that these properties observed *in vitro* for the Ornstein Uhlenbeck drive could be generalized to other type of input correlations.

In the time domain, the threshold model predicts an instantaneous response to step-like stimuli. Yet surprisingly, early experimental evidence presented by Silberberg and colleagues did not support the presence of an instantaneous response component [14]. However, this study did not quantify the response time scale and its conclusions might be biased by the use of a 4AP induced noise of unspecified correlation time or an almost white noise background, which in theoretical studies has been shown to lack an instantaneous component. The lack of rapid population response to step input, on the one side, and remarkably broad range of encoded frequencies on the other side presented an apparent contradiction. A recent study, however, has confirmed the broad reliably encoded frequency range *in vitro* and *in vivo* and has shown that in the presence of correlated noise cortical neurons *in vitro* can detect the step onset within milliseconds after its onset for a variety of conditions [25]. Also, a recent *in vivo* study by London and colleagues reported remarkably fast detection of subtle 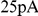 mean current pulses into a single neuron in the local cortical circuit *in vivo* [56]. This rapid response onset and the broad reliably encoded frequency range *in vitro* and *in vivo* that depends on the correlation time of the background can be understood using the theoretical results obtained here in a threshold model framework. Let us also note that the pairwise spike synchrony properties derived from the mean response function in the threshold framework, such as the firing rate dependence of correlation gain, are consistent with the experimental observations *in vivo* and *in vitro* [5], [38], [42]. For variance-encoded signals, the only experimentally study by Boucsein and colleagues reported a remarkably broad range of encoded frequencies *in vitro* [24]. The ability to rapidly detect the step-like change of input current variance in a population of neurons has, however, been shown only for large changes of variance (for doubling [14] or tripling [25] of the input current variance). In summary, while the existence of a fast response component in response to step-like variance changes could be confirmed *in vitro* it may require a larger amplitude than predicted by this threshold model. This could be due to additional cellular threshold adaptation mechanisms or voltage dependent conductance changes-effects that are not included in this threshold model framework.

### Outlook and novel model predictions

Here, we have shown that an alternative threshold model framework can capture many important features of the frequency response functions *in vivo* and *in vitro*. Importantly, this framework also offers novel predictions that can be tested experimentally. One important new prediction is the independence of the mean evoked response function on the functional form of the input correlations. In Eq. 21 the mean evoked response function depends only on the width 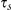 of the input correlation in the vicinity of zero, but not on any other functional specifics. For live neurons, this prediction means that any change in ion channel composition that affects the periphery rather than the central peak of the correlation function will not affect the frequency response function. This prediction can be directly tested *in vitro* with two current correlation functions which share the same 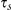 but have otherwise very different functional forms. On the theoretical side, we provided a number of tractable expressions for the complete spike triggered average and complete linear pairwise spike correlation function, and mean as well as variance evoked response functions. This analytical tractability framework will make it a useful addition to the theoretical toolkit of integrate and fire models and facilitate future model-model as well as model-experiment comparisons.

## Methods

### Current mean and variance in a cortical network

According to shot noise theory [57], the population average of the total, network generated input to a neuron in the kth population, e.g. where k = 1 stands for the excitatory and k = 2 for the inhibitory population is characterized by its mean 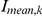 and variance 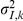. These are:
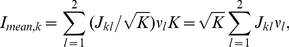
(40)

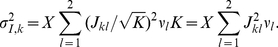
(41)Here, each spike evokes a postsynaptic current pulse described by the function 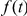 which is normalized 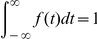, 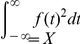. 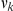 is the population averaged firing rate for neurons of type 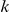, 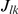 is the unit connection strength, 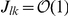. The connection strength between two neurons of type 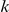 and 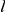 is 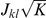 with probability 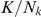 and zero otherwise. On average, K excitatory and K inhibitory neurons project to each neuron and the total number of neurons 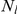 of any type 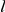 is large with regard to 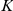, 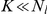. Let us note that an excellent derivation of the above equations for a balanced network can be also found in [58].

For the sake of tractability, let us focus on 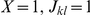 and denote the excitatory rate by 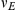 and inhibitory by 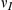. We obtain the mean and variance for all neurons in the network:
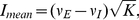
(42)

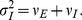
(43)We use these simplified equations in Section “Signal representation in cortical networks” to investigate two statistically independent ways to encode a signal in a population of cortical neurons.

### Mean modulation

Here we calculate the full response function for mean modulating signals. First, the membrane filtered signal 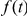 from Eq. 19
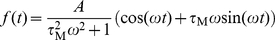
(44)is added to the membrane potential 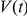 and offsets the threshold in the spike train 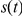:
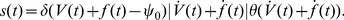
(45)Solving for the periodically modulated population firing rate we obtain:
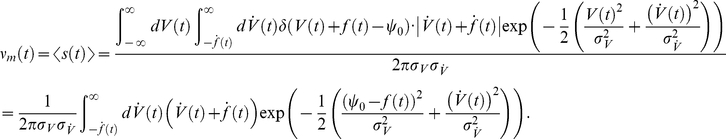
(46)This integral can be solved analytically using standard Gaussian integrals. It yields the complete response function given in Eq. 20.

### Variance modulation

Here we calculate the voltage correlations 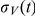, 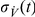 and the cross correlation 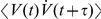 which we use in Eq. 24 to express the population firing rate response to oscillatory variance modulations. We start with the definition of the periodic current variance modulation and the resulting voltage 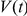:
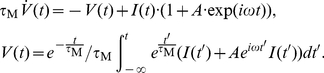
(47)To calculate the firing rate modulation we need to choose a current correlation function. We choose 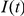 to be the Ornstein-Ulenbeck current and the current correlation function 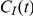 to be an exponential with correlation time 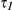, as in previous leaky integrate and fire studies. For this functional choice all integrals are analytically solvable. First, we address 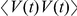:
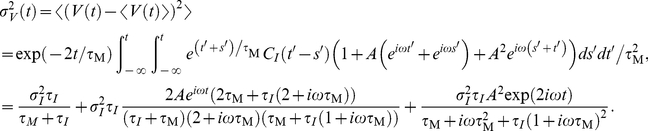
(48)Let us now address 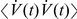


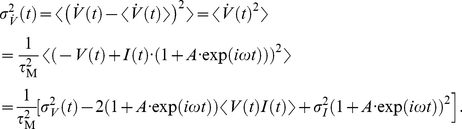
(49)Using 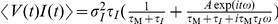 we obtain
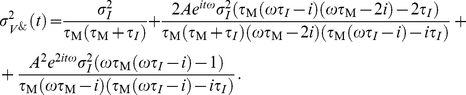
(50)We now address 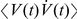:
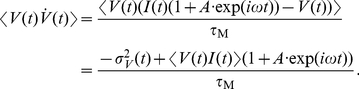
(51)Taking 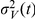 and 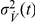 allows us to calculate the firing rate 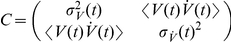


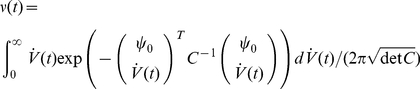
(52)Solving this integral using standard Gaussian integrals and covariances obtained above we obtain Eq. 23.

### Quantification of oscillatory firing rate modulations

**Figure 9:**
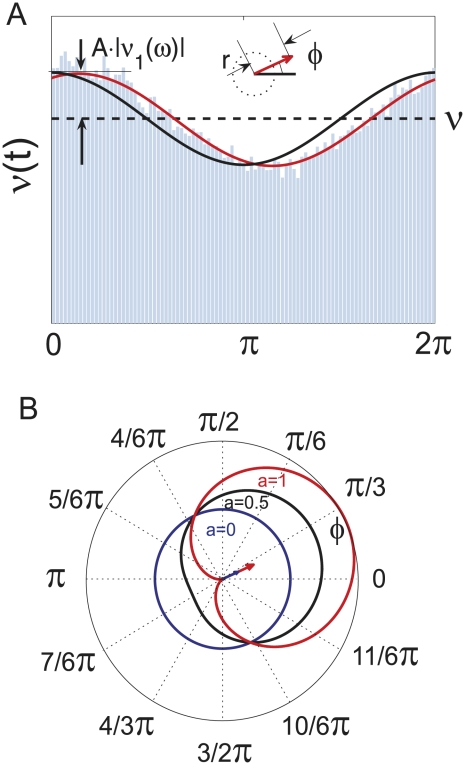
Demonstration of population firing rate modulation and phase locking. (A) Simulated population firing rate 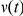 for mean current modulation for 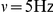, 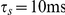, 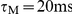 and 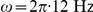, time bin 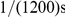. This results in 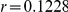, 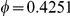 and in the amplitude of the firing rate modulation of 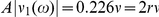. Solid lines denote the envelop of 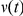 (red) and the current modulation (black). Black and red arrows indicate the phase relation between the input current and the evoked firing rate response. (B) Theoretical distribution of phase lags 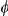 for varying modulation depth 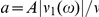, for illustration we chose 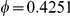 (from (A)). The solid curves are the distribution envelop for 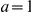 (red), 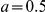 (black), 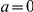 (blue). The arrows indicate the corresponding mean phase 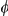.

We use the vector strength 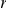 [8] to obtain the linear response function numerically:
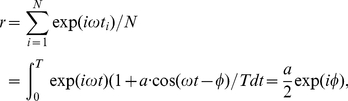
(53)where 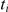 are the spike times, 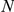 their number, 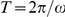 is the period length and 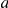 is the relative amplitude of the firing modulation evoked by the signal 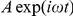. Taking 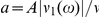 we can directly identify the vector strength as 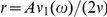. Here 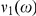 represents either 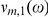 or 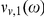. Fig. 9 illustrates the relation between the linear response function 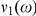 and the vector strength 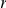. Because the vector strength 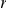 is constructed directly from the spike times, it omits a sinusoid fit of the peristimulus time histogram, which can potentially be biased by the fitting algorithm or the bin size chosen.

### Spike triggered average voltage

Here, we obtain the spike triggered average voltage 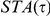 a time lag 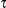 before a spike in the threshold model via a direct calculation of Gaussian integrals:
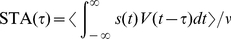
(54)

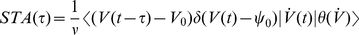
(55)

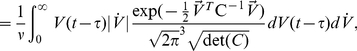
(56)where the correlation matrix is
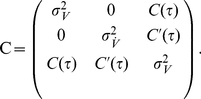
(57)Using standard Gaussian integrals we obtain the result in Eq. 37, which reads:
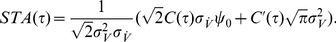
(58)


## References

[1] Volgushev M, Pernberg J, Eysel UT (2003). γ-frequency fluctuations of the membrane potential and response selectivity in visual cortical neurons.. Eur J Neurosci.

[2] Dayan P, Abbott LF (2001). Theoretical Neuroscience: Computational and Mathematical Modeling of Neural Systems.

[3] Buzsaki G, Draguhn A (2004). Neuronal oscillations in cortical networks.. Science.

[4] Riehle A, Gruen S, Diesmann M, Aertsen A (1997). Spike synchronization and rate modulation differentially involved in motor cortical function.. Science.

[5] Greenberg DS, Houweling AR, Kerr JND (2008). Population imaging of ongoing neuronal activity in the visual cortex of awake rats.. Nat Neurosci.

[6] Margrie TW, Brecht M, Sakmann B (2002). In vivo, low-resistance, whole-cell recordings from neurons in the anaesthetized and awake mammalian brain.. Pflugers Arch.

[7] Knight B (1972). Dynamics of encoding in a population of neurons.. J Gen Physiol.

[8] Goldberg JM, Brown PB (1969). Response of binaural neurons of dog superior olivary complex to dichotic tonal stimuli: some physiological mechanisms of sound localization.. J Neurophysiol.

[9] Lindner B, Schimansky-Geier L (2001). Transmission of noise coded versus additive signals through a neuronal ensemble.. Phys Rev Lett.

[10] Brunel N, Chance F, Fourcaud N, Abbott LF (2001). Effects of synaptic noise and filtering on the frequency response of spiking neurons.. Phys Rev Lett.

[11] Fourcaud-Trocme N, Hansel D, van Vreeswijk CA, Brunel N (2003). How spike generation mechanisms determine the neuronal response to fluctuating inputs.. J Neurosci.

[12] Naundorf B, Geisel T, Wolf F (2005). Action potential onset dynamics and the response speed of neuronal populations.. J Comput Neurosci.

[13] Gerstner W (2000). Population Dynamics of Spiking Neurons: Fast Transients, Asynchronous States, and Locking.. Neur Comp.

[14] Silberberg G, Bethge M, Markram H, Pawelzik K, Tsodyks M (2004). Dynamics of population rate codes in ensembles of neocortical neurons.. J Neurophysiol.

[15] Ostojic S, Brunel N, Hakim V (2009). How connectivity, background activity, and synaptic properties shape the cross-correlation between spike trains.. J Neurosci.

[16] Ostojic S, Brunel N (2011). From spiking neuron models to linear-nonlinear models.. PLoS Comput Biol.

[17] Alijani A, Richardson M (2011). Rate response of neurons subject to fast or frozen noise: From stochastic and homogeneous to deterministic and heterogeneous populations.. Phys Rev E.

[18] Brunel N, Latham P (2003). Firing rate of the noisy quadratic integrate-and-fire neuron.. Neural Comput.

[19] Fourcaud-Trocme N, Brunel N (2005). Dynamics of the instantaneous firing rate in response to changes in input statistics.. J Comput Neurosci.

[20] Wei W, Wolf F (2011). Spike Onset Dynamics and Response Speed in Neuronal Populations.. Phys Rev Lett.

[21] Pressley J, Troyer TW (2011). The dynamics of integrate-and-fire: Mean versus variance modulations and dependence on baseline parameters.. Neuro Comp.

[22] Pressley J, Troyer TW (2009). Complementary responses to mean and variance modulations in the perfect integrate-and-fire model.. Biol Cybern.

[23] Koendgen H, Geisler C, Fusi S, Wang XJ, Luescher HR (2008). The dynamical response properties of neocortical neurons to temporally modulated noisy inputs in vitro.. Cereb Cortex.

[24] Boucsein C, Tetzlaff T, Meier R, Aertsen A, Naundorf B (2009). Dynamical response properties of neocortical neuron ensembles: Multiplicative versus additive noise.. J Neurosci.

[25] Tchumatchenko T, Malyshev A, Wolf F, Volgushev M (2011). Ultra-fast population encoding by cortical neurons.. J Neurosci.

[26] Destexhe A, Rudolph M, Pare D (2003). The high-conductance state of neocortical neurons in vivo.. Nat Rev Neurosci.

[27] Zito K, Scheuss V, Squire L (2007). NMDA Receptor Function and Physiological Modulation.. The New Encyclopedia of Neuroscience.

[28] Lampl I, Reichova I, Ferster D (1999). Synchronous membrane potential fluctuations in neurons of the cat visual cortex.. Neuron.

[29] Shadlen MN, Newsome WT (1994). Noise, neural codes and cortical organization.. Curr Opin Neurobiol.

[30] Okun M, Lampl I (2008). Instantaneous correlation of excitation and inhibition during ongoing and sensory-evoked activities.. Nat Neurosci.

[31] Renart A, de la Rocha J, Bartho P, Hollender L, Parga N (2010). The asynchronous state in cortical circuits.. Science.

[32] Ecker AS, Berens P, Keliris GA, Bethge M, Logothetis NM (2010). Decorrelated neuronal firing in cortical micro circuits.. Science.

[33] Peters A, Payne B, Budd J (1994). A numerical analysis of the geniculocortical input to striate cortex in the monkey.. Cereb Cortex.

[34] Wiener N (1958). Nonlinear problems in random theory.

[35] Jung P (1995). Stochastic resonance and optimal design of threshold detectors.. Phys Lett A.

[36] Burak Y, Lewallen S, Sompolinsky H (2009). Stimulus-dependent correlations in threshold-crossing spiking neurons.. Neur Comput.

[37] Tchumatchenko T, Geisel T, Volgushev M, Wolf F (2010). Signatures of synchrony in pairwise count correlations.. Front Comput Neurosci.

[38] Tchumatchenko T, Malyshev A, Geisel T, Volgushev M, Wolf F (2010). Correlations and synchrony in threshold neuron models.. Phys Rev Lett.

[39] Prichard D, Theiler J (1994). Generating surrogate data for time series with several simultaneously measured variables.. Phys Rev Lett.

[40] Schneidman E, Berry MJ, Segev R, Bialek W (2006). Weak pairwise correlations imply strongly correlated network states in a neural population.. Nature.

[41] Ohiorhenuan I, Mechler F, Purpura KP, Scmid A, Hu Q (2010). Sparse coding and high-order correlations in fine-scale cortical networks.. Nature.

[42] de la Rocha J, Doiron B, Shea-Brown E, Josić K, Reyes A (2007). Correlation between neural spike trains increases with firing rate.. Nature.

[43] Shea-Brown E, Josić K, de la Rocha J, Doiron B (2008). Correlation and synchrony transfer in integrate-and-fire neurons: Basic properties and consequences for coding.. Phys Rev Lett.

[44] Mainen ZF, Sejnowski TJ (1995). Reliability of spike timing in neocortical neurons.. Science.

[45] Badel L, Gerstner W, Richardson M (2008). Spike-triggered averages for passive and resonant neurons receiving filtered excitatory and inhibitory synaptic drive.. Phys Rev E Stat Nonlin Soft Matter Phys.

[46] Aguera y Arcas B, Fairhall AL (2003). What causes a neuron to spike?. Neural Comput.

[47] Schwartz O, Pillow JW, Rust NC, Simoncelli EP (2006). Spike-triggered neural characterization.. J Vision.

[48] Gazzaniga MS (2004). Cognitive neuroscience III.

[49] Tchumatchenko T, Geisel T, Volgushev M, Wolf F (2011). Spike correlations-what can they tell about synchrony?. Front Neurosci.

[50] Naundorf B, Wolf F, Volgushev M (2006). Unique features of action potential initiation in cortical neurons.. Nature.

[51] Badel L, Lefort S, Brette R, Petersen CCH, Gerstner W (2008). Dynamic I–V Curves Are Reliable Predictors of Naturalistic Pyramidal-Neuron Voltage Traces.. J Neurophysiol.

[52] Vilela RD, Lindner B (2009). Comparative study of different integrate-and-fire neurons: Spontaneous activity, dynamical response, and stimulus-induced correlation.. Phys Rev E.

[53] Knight BW (1972). The relationship between the firing rate of a single neuron and the level of activity in a population of neurons.. J Gen Physiol.

[54] Carandini M, Mechler F, Leonard CS, Movshon JA (1996). Spike train encoding by regular-spiking cells of the visual cortex.. J Neurophysiol.

[55] Bair W, Koch C (1996). Temporal precision of spike trains in extrastriate cortex of the behaving macaque monkey.. Neural Comp.

[56] London M, Roth A, Beeren L, Häusser M, Latham PE (2010). Sensitivity to perturbations in vivo implies high noise and suggests rate coding in cortex.. Nature.

[57] van Kampen NG (2007). Stochastic Processes in Physics and Chemistry..

[58] van Vreeswijk CA, Sompolinsky H (1998). Chaotic balanced state in a model of cortical circuits.. Neural Comp.

